# Dynamic Compression Improves Chondrogenesis in the Tissue Engineered Model of Cartilage

**DOI:** 10.1002/bit.29026

**Published:** 2025-05-25

**Authors:** Marc V. Farcasanu, Thais de las Heras Ruiz, Francesca M. Johnson de Sousa Brito, Jamie Soul, Jonathan Coxhead, Matthew J. German, David A. Young, Ana M. Ferreira‐Duarte, Katarzyna A. Piróg

**Affiliations:** ^1^ Biosciences Institute Newcastle University Newcastle upon Tyne UK; ^2^ Computational Biology Facility University of Liverpool Liverpool UK; ^3^ School of Dental Sciences Newcastle University Newcastle upon Tyne UK; ^4^ School of Engineering Newcastle University Newcastle upon Tyne UK

**Keywords:** cartilage development, dynamic compression, hydrogel, tissue engineering

## Abstract

Hyaline cartilage is a dense avascular tissue with low regenerative potential, present at the ends of the diarthrodial joints and in the cartilage growth plate. Skeletal diseases often result from extracellular changes in this tissue; however, studies of these are hindered by the tissue complexity, the difficulty in obtaining human material, and the cost of generating animal models. Recent developments in tissue engineering are opening possibilities to develop mechanoresponsive zonally stratified models of cartilage in vitro. In this study, we optimized a 3D model of cartilage using chondroprogenitor cells cultured for 21 days in 2% agarose hydrogel constructs with daily dynamic compression. Our hydrogel constructs developed pericellular matrices with nanostiffness comparable with native murine tissue and showed increased production of extracellular matrix components and expression of chondrogenic and differentiation markers. Daily dynamic compression resulted in progressive increase in mechanoresponsive gene expression and promoted a juvenile cartilage phenotype, decreasing expression of dedifferentiation and cartilage degradation markers. Our study highlights the potential of hydrogel‐enhanced chondrogenesis and proposes an adaptable and scalable in vitro model to study mechanoresponses, intracellular signals, and pericellular matrix involvement in cartilage development and disease.

## 1 Introduction

Cartilage tissue engineering remains a challenge due to the complex zonally stratified structure of hyaline cartilage combined with necessity to provide dense extracellular environment with specific mechanical properties. Moreover, cartilage is an avascular tissue with low regenerative potential, and obtaining primary cells from healthy cartilage samples is hindered by tissue availability (Kisiday 2020). ATDC5 cells, a chondrogenic cell line derived from mouse teratocarcinoma AT805, are commonly used to model cartilage development and to screen novel genetic variants of cartilage disease thanks to their high proliferation rates, and ability to recapitulate chondrogenesis in monolayer, from mesenchymal condensation to calcification (Newton et al. 2012; Wilhelm et al. 2020; Yao and Wang 2013). Upon stimulation with insulin and transferrin (Okita et al. 2023; Wen et al. 2023) and with the addition of ascorbic acid to promote collagen deposition (Okita et al. 2023), ATDC5 cells grown on tissue culture plastic upregulate chondrocyte specific gene expression and deposit extracellular proteoglycans. A relatively straightforward 2D culture of ATDC5 cells provides a useful model to study the effect of disease‐causing variants or gene silencing on chondrogenic differentiation (Dong et al. 2021; Hodax et al. 2019; Xu et al. 2019). ATDC5 cells can also be used to study calcification whereby organic phosphate is added to the cultures to promote matrix mineralization (Newton et al. 2012; Tschaffon et al. 2021; Wu et al. 2017). However, 2D culture leads to the absence of zonal stratification typically found in hyaline cartilage, as mature chondrocyte phenotypes are often induced by depleted access to nutrients and changes in oxygen tension (Silva et al. 2020).

To date, most tissue engineering studies using chondrocytes focused on relatively small micromass and alginate bead constructs or the use of foams and sponges as surface support structures. The importance of three‐dimensional environment for the differentiation of ATDC5 cells has been highlighted by the studies whereby cells were grown in micromass and in alginate beads, both of which improved their chondrogenic potential (Lewis et al. 2016; Takeda et al. 2021). In this paper we specifically focused on optimizing supportive growth factor supplementation to promote early ATDC5 chondrogenesis in larger pellet cultures, and on engineering a hydrogel‐based model of hyaline cartilage that could be cultured under dynamic compression. The 2% low melting agarose has previously been shown to support maintenance of human primary chondrocytes and to promote mesenchymal stem cell differentiation (Charles Huang et al. 2004; Thompson et al. 1985). Moreover, it has been shown that construct constriction with a free‐swelling surface creates a gradient in oxygen tension that supports chondrogenic differentiation of mesenchymal stem cells (Thorpe et al. 2013). Cartilage is a mechanoresponsive tissue and dynamic compression interspersed with periods of rest is beneficial for its homeostasis (Nicodemus and Bryant 2010). Compressive regimes that resemble physiological stimuli have been shown to support skeletal phenotypes, whereas excessive load leads to increased inflammation signals and markers such as *IL1R*, reminiscent of osteoarthritis (Choi et al. 2018; Nicodemus and Bryant 2010; Thorpe et al. 2010). In fact, many cartilage specific genes are mechanoresponsive (Watts et al. 2013) and proteoglycans and other extracellular matrix (ECM) components contribute to mechanical properties and act as signaling mediators of cellular processes such as adhesion, cell proliferation, and differentiation (Silva et al. 2020).

In our study, we used the 3D pellet system to test the effects of BMP7, TGFβ3, and combination of both growth factors on ATDC5 chondrogenesis. We then used the improved growth factor supplementation to augment chondrogenic differentiation of ATDC5 chondroprogenitor cells embedded in 2% low melting agarose and investigated the effect of repetitive sinusoid dynamic compressive strain on chondrogenesis (Figure S1A,B). We show that chondrogenic differentiation of ATDC5 cells can be improved by an optimized growth factor supplementation, that 2% agarose supports chondrogenic differentiation of ATDC5 cells and ECM production, and that our compressive regime supports a juvenile chondrocyte phenotype. The easy to manufacture agarose‐based constructs seeded with commercially available ATDC5 cells show markers of cartilage differentiation and changes in mechanoresponses, providing a model that can be applied to study mechanical responses and for future preclinical screening of therapeutic compounds.

## 2 Materials and Methods

### 2.1 Cell Culture

ATDC5 cells (ECACC 99072806) were cultured in Dulbecco's Modified Eagle's Medium (DMEM; Gibco™ 11995073) supplemented with 10% fetal calf serum (FCS; Sigma‐Aldrich) and 1% non‐essential amino acids (NEAA; Gibco™) at 5% CO_2_ and 37°C, then seeded at 6000 cells/cm^2^ on plastic, and at 500,000 cells per pellet. The cells were then grown in chondrogenic medium consisting of DMEM/F12 (Gibco™ 10565018) supplemented with 5% FCS, 1% NEAA, 1% sodium pyruvate (Gibco™), 1% insulin‐transferrin‐selenium (ITS; Sigma‐Aldrich), and 50 μg/mL ascorbic acid (Sigma‐Aldrich) from Day 6 after 2D plating as per standard ATDC5 chondrogenesis protocol (Newton et al. 2012), with the following day marked as “Day 1” in the analysis. ITS and ascorbic acid were added to medium before use.

### 2.2 Pellet Culture

5 × 10^5^ ATDC5 cells were centrifuged in universal tubes for 10 min at 8000*g*. Media was changed to the chondrogenic medium with or without growth factor supplementation the following day (Day 1) with gentle pipetting to increase the rotation and the rounded shape of the pellet. Medium was changed every 2 days with supplements (ITS, ascorbic acid) and growth factors (10 ng/mL of BMP7 (Peprotech 120‐03P) and/or 10 ng/mL TGFβ3 (Peprotech 100‐36E)) added freshly before use.

### 2.3 Agarose Constructs

To generate the agarose constructs, 2.5% w/v low gelling agarose (Sigma‐Aldrich A9045) was prepared and sterilized by autoclaving. The agarose was melted in an oven set to 60°C and cooled to 39°C in a water bath before use. 10 × 10^6^ ATDC5 cells/mL were mixed with medium and with the agarose to the final concentration of 2% w/v low gelling temperature agarose, poured into sterilized stainless‐steel molds placed in standard 12‐well tissue culture plates and allowed to set for 5 min in a sterile laminar flow hood before chondrogenic medium was added. The tissue shapes were uniform across the experiments, standardized by ensuring the correct agar concentration, gelation time, and temperature, and were consistently 3 mm in height. Medium was changed every 2 days with supplements (ITS, ascorbic acid, and growth factors (10 ng/mL of BMP7 and/or 10 ng/mL TGFβ3) added freshly before use.

### 2.4 Dynamic Compression

Dynamic compression was applied to the agar constructs only. Compression was applied on Day 7 postseeding using the pneumatic Flexcell FX5000™ Compression System (Dunn Labortechnik GmbH). Briefly, constructs were transferred to the sterile BioPress™ (Dunn Labortechnik GmbH) six‐well culture plates. To avoid preloading the samples, stationary platens placed into the wells were tightened into place as per manufacturer's protocol to bring the bottom of the center screw to the point where it just touched the top of the sample. Compression was applied for 30 min daily at 10 kPa, 0.33 Hz sinusoid regime, performed at the same time of day for all constructs to account for circadian rhythm. The location of the individual plates on the baseplate was randomized. Medium was changed every 2 days using the holes around the inner periphery of the platen, with supplements (ITS, ascorbic acid, and growth factors (10 ng/mL of BMP7 and/or 10 ng/mL TGFβ3) added freshly before use. The constructs were allowed to recover for 1 h before harvest.

### 2.5 Scanning Electron Microscopy (SEM)

Agarose constructs were washed with PBS and fixed in 2% glutaraldehyde in Sorenson's Phosphate Buffer (VWR) overnight, then rinsed and dehydrated in increasing grades of ethanol. Samples were then dried completely using a Baltec CPD 030 Critical Point Dryer. A 15 nm coating of gold was performed using a Polaron SEM Coating Unit after which samples were mounted on an aluminum stub with carbon discs. A Tescan Vega3LMU scanning electron microscope was used to acquire the images (Tescan). False coloring was performed in Adobe Photoshop 26.4.1.

### 2.6 Atomic Force Microscopy (AFM)

AFM measurements were performed using NanoWizard®3 (JPK Instruments). The hydrogel constructs were immersed in 1x PBS during measurements. Triangular probes (Bruker) with a nominal spring constant of 0.35 N/m and a nominal tip radius of 20 nm were utilized. Measurements were taken in a 25 × 25 µm scan area at a resolution of 128 × 128 pixels in four separate areas were probed within a 25 µm offset in both the *x*‐ and *y*‐axis. Young's Modulus was calculated based on Hertz model:

$$F=\frac{2\tan\left(\alpha \right)E}{\pi (1-\upsilon^{2})}\delta^{2}$$

where *α* is the pyramid angle (17.5° for the cantilever used), *E* is the Young's modulus, *υ* is the Poisson's ratio (assumed to be 0.5), and *δ* is the indentation depth. The extend part of the force‐indentation curves was used to obtain the Young's modulus values.

### 2.7 Histology and Immunohistochemistry

Three‐Dimensional cultures (pellets and hydrogels) were fixed in 4% paraformaldehyde (PFA, Sigma‐Aldrich), wax embedded and cut at 5 µm thickness. Samples were dewaxed, rehydrated, and every 5th slide was stained with 0.04% Toluidine blue (w/v, Sigma‐Aldrich) in 0.1 M acetate buffer (pH 4) to match the sections. Haematoxylin and Eosin (general morphology, Abcam), 1% w/v Alcian Blue in 0.1 M HCl (Sigma Aldrich) and Toluidine Blue (proteoglycans), and Picrosirus Red (collagen, Abcam) stains were performed on sections from matched regions of the samples to enable direct comparison. Nuclear Fast Red (Vector Labs) was used as nuclear counterstain. Immunohistochemistry was carried out as described previously (Pirog‐Garcia et al. 2007), with DAPI as nuclear counterstain (Abcam). The DeadEnd™ Fluorometric TUNEL System (Promega) was used to quantify apoptosis. Histological sections of all agar constructs were imaged with the free‐swelling part of the construct at the top of the image. All images were acquired using an Axioscan Z1 slide scanner (Zeiss). Cell free hydrogels were used as negative controls.

### 2.8 DMMB Assay

Samples were digested with papain (Sigma‐Aldrich P4762) at 60°C for 12–16 h and the 1,9‐dimethylmethylene blue (DMMB, Sigma) assay was used to measure sulfated glycosaminoglycans (sGAG) with Chondroitin sulfate A sodium salt from bovine trachea (Sigma‐Aldrich 230699) as standard (Farndale et al. 1986). GAG measurements were normalized to the DNA content. Briefly, samples were incubated at 70°C water bath to liberate DNA, vortexed briefly, then centrifuged at 10,000*g* for 5 min to pellet remaining debris. Digested samples in triplicates and DNA standards of known concentration were mixed 1:10 v/v with 0.2 µg/mL Hoechst 33258 (Sigma‐Aldrich) in TNE, and the fluorescence was measured at 360 nm excitation and 460 nm emission in a Varioskan LUX Multimode Microplate Reader (Thermo Scientific™). Cell free hydrogels were used as negative controls.

### 2.9 MTT Assay

The MTT (4,5‐dimethylthiazol‐2‐yl‐2,5‐diphenyltetrazolium bromide, Sigma‐Aldrich) assay was used to assess metabolic activity of the cells. Briefly, MTT was mixed with media 1:4 and incubated with the cells for 24 h. The resulting formazan crystals were solubilized in dimethyl sulfoxide (DMSO, Sigma‐Aldrich) and measured at 550 nm using the Varioskan LUX Multimode Microplate Reader (Thermo Scientific™). Agarose hydrogels were dehydrated and the remaining crystals were solubilized in DMSO then analyzed.

### 2.10 Quantitative RT‐PCR

RNeasy® mini kit (QIAgen) was used to extract the RNA from the 3D models. The manufacturer's protocol was modified as per protocol developed for agarose gels by Bougault et al. (2009). Briefly, pellets were snap frozen in RLT buffer (QIAgen) with β‐mercaptoethanol (Sigma‐Aldrich) using liquid nitrogen, then homogenized in the Mikro‐Dismembrator S (Sartorius) homogenizer for 2 min at 1000 rpm. Three‐Dimensional agarose cultures were dissolved in 2 mL RLT buffer with 1.5 mL QG buffer (QIAgen) and 2 μL β‐mercaptoethanol, and passed through a QIAshredder® (QIAgen) column before RNA extraction (RNeasy® mini kit (QIAgen)). GoScript™ Reverse Transcription System (Promega) was used to generate cDNA which was RNase H (Sigma‐Aldrich) treated and subject to quantitative real‐time PCR using Power® SYBR™ Green PCR Master Mix (ABI) (primers in Table S1). Results were analyzed using the $2^{‐∆∆C_{t}}$ method (Livak and Schmittgen 2001).

### 2.11 RNA Sequencing

RNA from three biological replicates was subject to RNase‐free DNAseI treatment (EN0523, Fisher Scientific) and TruSeq Stranded mRNA Sequencing – NextSeq High‐output 75 bp single read at an average 19 M reads per sample (Gene Expression Omnibus (GEO) accession number GSE280260). Quality control (QC) was performed using FastQC (v0.11.9). Reads were quality trimmed with Trimmomatic (0.39). Kallisto (v0.46.1) was used for pseudoalignment against Ensembl mouse GRCm38 (release 94) transcriptome. Mapped transcript expression estimates were summarized to the gene level using Tximport (v1.14.0) and DESeq. 2 (v1.26.0) was used to calculate log2 fold‐change (logFC) and *p* values using the Wald test and subsequently corrected for multiple testing with the Benjamini and Hochberg method. Pathway and gene ontology analysis was performed using differentially expressed genes defined as absolute fold change ≥2 and ≤5% False Discovery Rate (FDR) using Database for Annotation, Visualization and Integrated Discovery (DAVID) software (Huang et al. 2009).

### 2.12 Statistical Analysis

Power calculations were performed before the experiments to determine the sample size using G*Power 3.1 software (Universität Kiel, Germany; Faul et al. 2007). Statistical significance was determined using Student's two‐tailed *t* test for single comparisons or analysis of variance (ANOVA) for testing the effects in multiple samples. Statistical significance was determined at *p* < 0.05. All data analysis and statistical calculations were performed using the GraphPad Prism v10.2.3 software (GraphPad Software LLC).

## 3 Results

### 3.1 Growth Factor Supplementation Improves Chondrogenic Differentiation of ATDC5 Cells in Pellets

**Figure 1:**
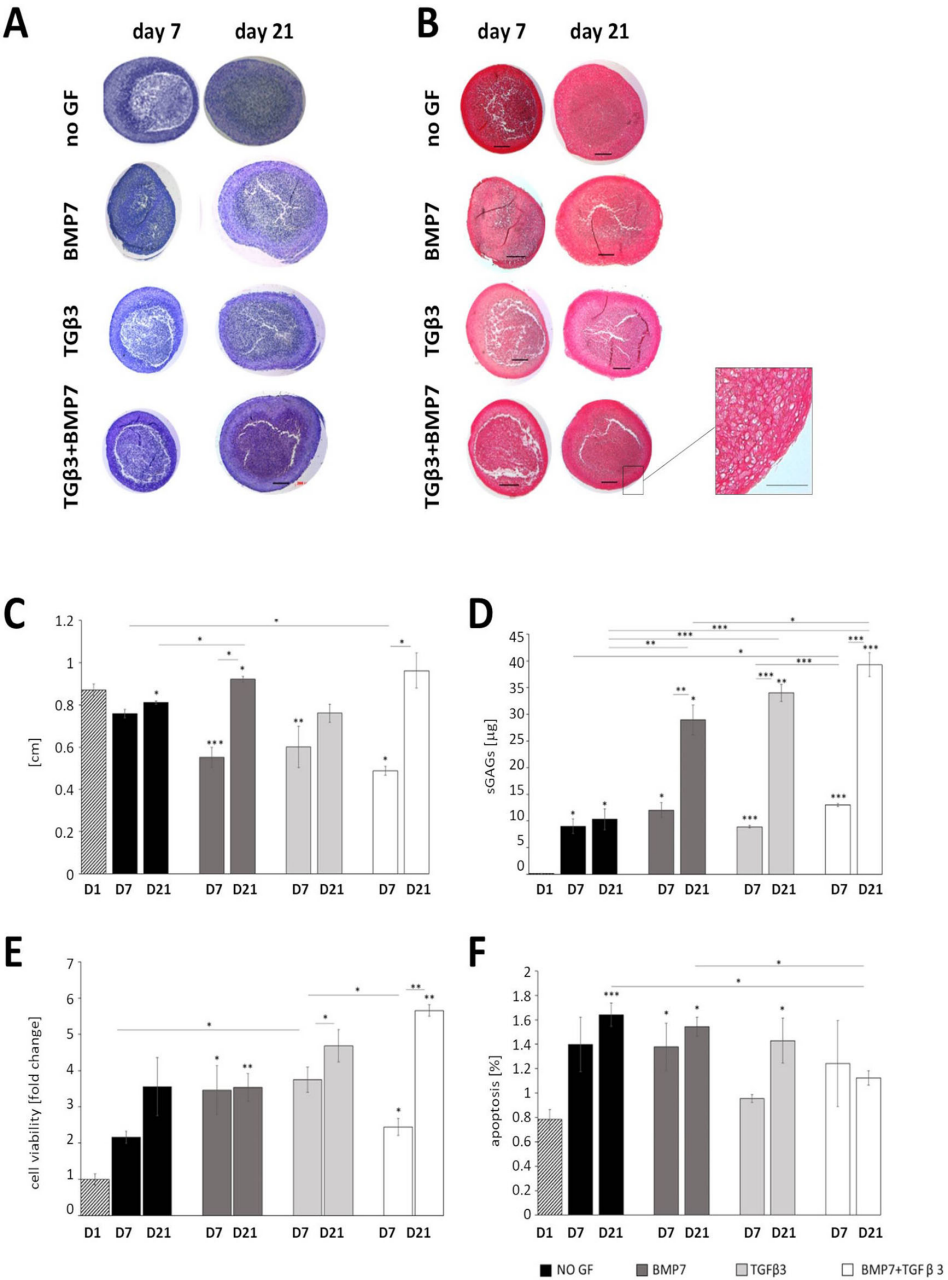
(A) Toluidine Blue staining showing proteoglycan deposition over 21 days of culture in the ATDC5 pellets cultured in the chondrogenic medium with growth factor supplementation, showing increased proteoglycan staining in pellets cultured with a combination of growth factors at Day 21. (B) Picrosirus Red staining for collagen deposition in ATDC5 pellets grown for 21 days with and without growth factor supplementation, showing uniform collagen production in all cultures. Inset: pellets show morphological zonal stratification with smaller flat cells on the pellet surface and larger cells deeper in the pellet. (C) Quantification of the area of ATDC5 pellets growth with different growth factor supplementation. (D) Quantification of the DMMB assay for sulphated proteoglycan deposition showing increased proteoglycan deposition in pelts cultured with growth factor supplementation, highest in pellets grown with a combination of TGFβ3 and BMP7. (E) Quantification of the MTT assay for cell metabolism performed for ATDC5 pellets grown with and without growth factor supplementation showing an increase in metabolic activity in pellets cultured with TGFβ3 and with TGFβ3 and BMP7. (F) TUNEL assay measuring apoptosis in the pellets, showing decreased cell death in pellets cultured with TGFβ3 and BMP7 over 21 days. Student *t* test, SEM, *n* = 3. Key: *p < 0.05; **p < 0.01; D7, Day 7; D21, Day 21. Scale bar 200 μm.

ATDC5 cells subjected to standard 2D chondrogenic differentiation protocol showed increased proteoglycan deposition over the 21 days of culture (Figure S1C) and increased expression of the main chondrogenic markers (*Sox9*, *Col2a1*, *Acan*, *Ihh*). However, the levels of expression of type I collagen (*Col1a1*) remained stable throughout the culture despite the lack of β‐glycerophosphate additive, indicating potential dedifferentiation or fibroblast/fibrocartilage‐like phenotype, consistent with the previous reports (Wilhelm et al. 2020) (Figure S1D–G). Therefore, to optimize our culture conditions, we cultured the ATDC5 cells for 21 days in pellets (Kim et al. 2022; Tare et al. 2005). Pellet culture improved the morphology of the cells which changed from fusiform to rounded (resembling the native chondrocytes) and resulted in deposition of cartilage‐like ECM rich in proteoglycans and collagens (Figure 1A,B). Pellet culture has been shown in the past to improve the differentiation of skeletal lineage cells (Tare et al. 2005; Watts et al. 2013; Wilhelm et al. 2020; Yao and Wang 2013), however, reports vary as to the most effective culture conditions (Caron et al. 2013; Lewis et al. 2016; Takeda et al. 2021; Tare et al. 2005; Weiss et al. 2012). Several growth factors have previously been tested in primary cells, with BMP7 shown to prevent terminal differentiation of chondrocytes, and TGFβ3 shown to increase ECM deposition of type II collagen in chondrocyte pellets (Caron et al. 2013; Huang et al. 2018). Moreover, it has been shown that combined treatment with 10 ng/mL TGFβ3 and/or 10 ng/mL BMP7 stimulates redifferentiation of dedifferentiated human primary chondrocytes (HACs) in both hypoxia and normoxia conditions (Huang et al. 2018). We therefore tested whether supplementation of the chondrogenic medium with 10 ng/mL of BMP7, TGFβ3 or with BMP7 and TGFβ3 combined could further improve ATDC5 chondrogenesis in the pellet system. Interestingly, supplementation with BMP7 or a combination of BMP7 and TGFβ3 together led to a steady increase in pellet size (Figure 1C). Proteoglycan deposition remained stable in unsupplemented pellets and increased from Day 7 to Day 21 of culture in all growth factor supplemented pellets (Figure 1D). Supplementation with TGFβ3 and with combined BMP7 and TGFβ3 increased metabolic activity of the cells (Figure 1E) and proliferation (Figure S2A). Apoptosis decreased in TGFβ3 and BMP7 supplemented pellets compared with unsupplemented pellets at Day 21 (Figure 1F).

**Figure 2:**
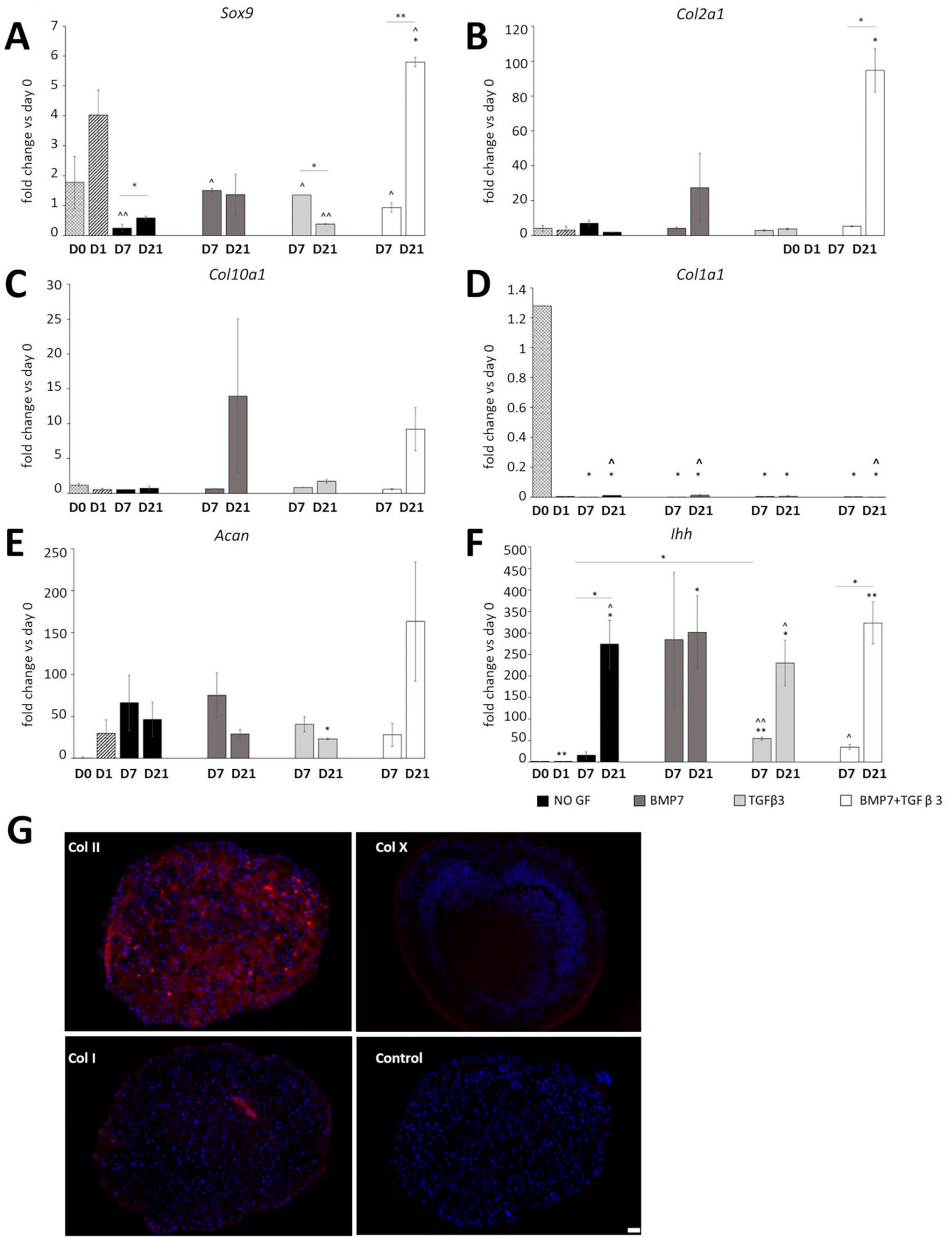
(A–F) qRT‐PCR for chondrogenesis (*Sox9*, *Col2a1*, *Acan*), differentiation (*Col10a1*, *Ihh*) and dedifferentiation (*Col1a1*) markers in 21‐day pellet culture of ATDC5 cells with growth factor supplementation (*n* = 3). (G) Immunohistochemistry for type II, type X, and type I collagen (in red) with DAPI nuclear counterstain (blue) in ATDC5 pellets cultured with TGFβ3 and BMP7 at 21 days. Student *t* test, SEM. Key: *p < 0.05 versus Day 0; **p < 0.01 versus Day 0; ^*p* < 0.05 versus Day 1; ^^*p* < 0.01 versus Day 1; NO GF, no growth factor supplementation; D7, Day 7; D21, Day 21. Scale bar 200 μm.

*Sox9* (transcription factor and a marker of chondrogenesis) expression was decreased at Day 7 in all supplemented pellets compared with Day 1 and increased by 2.4‐fold from Day 7 to Day 21 in unsupplemented pellets. Supplementation with TGFβ3 and BMP7 combined led to a dramatic increase in *Sox9* expression by Day 21 of culture compared with Day 0 (2D), Day 1 and Day 7 TGFβ3 and BMP7 supplemented control, while BMP7 alone had no effect on *Sox9* expression and TGFβ3 supplementation led to a 3.5‐fold decrease in expression at Day 21. TGFβ3 and BMP7 supplementation also resulted in an increase in *Col2a1* (type II collagen, the main collagen in cartilage) expression compared with Day 1 and with Day 7 TGFβ3 and BMP7 samples. *Col1a1* (type I collagen, marker of dedifferentiated cartilage) expression was decreased in all pellets compared with Day 0 2D controls and decreased in Day 7 pellets without supplementation, and in BMP7, and TGFβ3 and BMP7 supplemented pellets compared with Day 1. *Ihh*, involved in the regulation of chondrocyte proliferation and terminal differentiation, was increased in all pellets at Day 21 compared with 2D controls, increased from Day 7 to Day 21 of culture in pellets without growth factor supplementation, and in pellets supplemented with both growth factors (Figure 2A–F). Finally, immunohistochemistry showed deposition of type II collagen throughout the pellets supplemented with both growth factors, with type I collagen present in a thin layer on the surface of pellets at 21 days, potentially a result of shear stress during media changes (Figure 2G).

### 3.2 2% Agarose Supports Chondrogenic Differentiation of ATDC5 Cells

**Figure 3:**
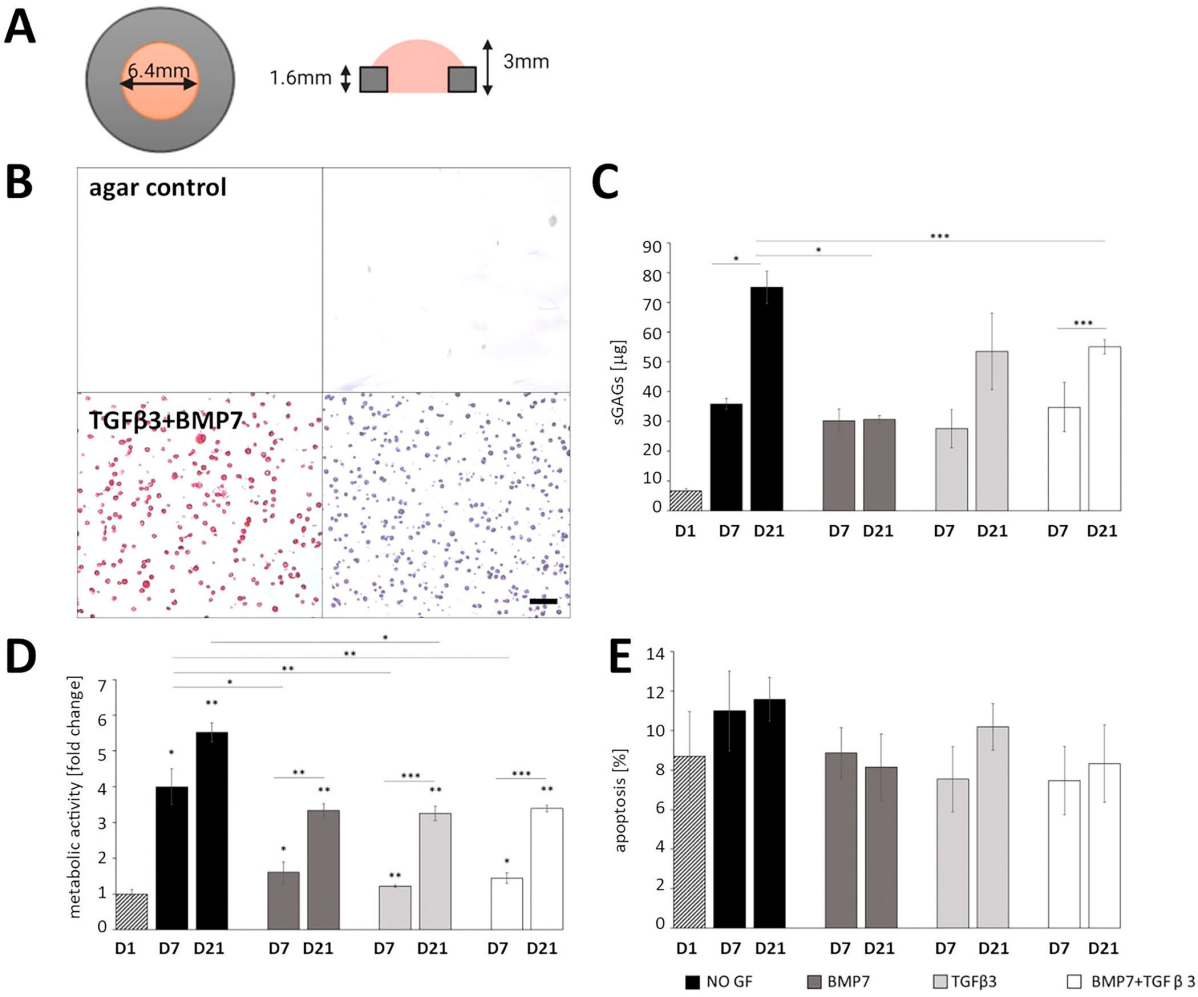
(A) Schematic image of the 2% low melting agarose construct, with the perimeter of the construct constricted by an autoclavable metal washer. (B) Histological assessment of the 2% agarose constructs with 10 × 10^6^ ATDC5 cells at Day 21 using Picrosirus Red (collagen deposition) and Toluidine Blue (proteoglycan deposition). (C) DMMB assay to quantify sulphated proteoglycans showing an increase in proteoglycan deposition in constructs cultured in chondrogenic medium alone and in constructs grown in the presence of TGFβ3 and BMP7 for 21 days (*n* = 3). (D) Metabolic activity of ATDC5 cells encased in agar measured by the MTT assay showing increased cell metabolic activity over time in all constructs cultured with growth factor supplementation (*n* = 3). (E) Quantification of the TUNEL assay for apoptosis, expressed as percentage of total cells in the tissue and showing that apoptosis is not increased by culture in low melting agarose and remained constant throughout the culture (*n* = 3). Student *t* test, SEM. Key: *p < 0.05; **p < 0.01; NO GF, no growth factor supplementation; D7, Day 7; D21, Day 21. Scale bar 100 μm.

**Figure 4:**
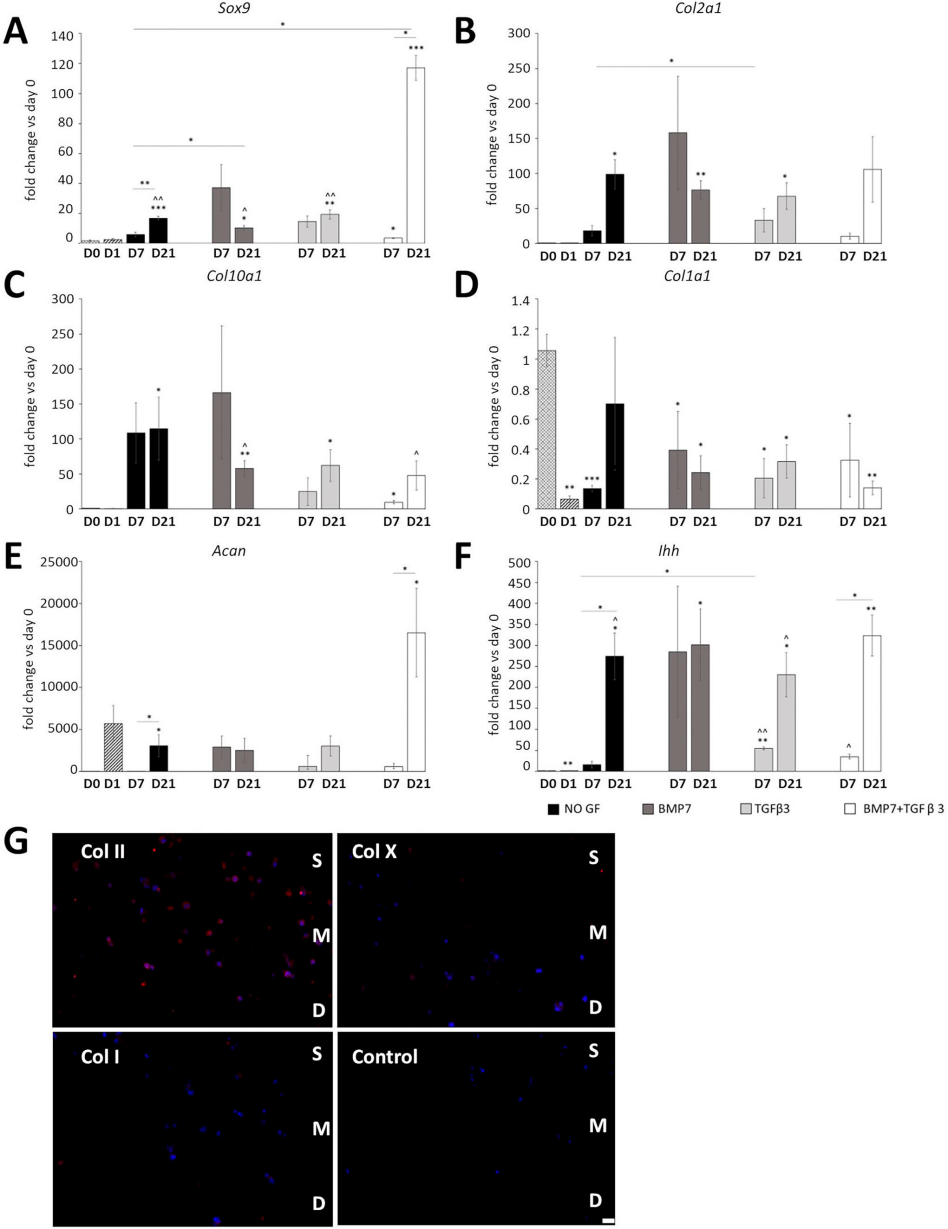
(A–F) qRT‐PCR of chondrogenesis (*Sox9*, *Col2a1*, *Acan*), differentiation (*Col10a1*, *Ihh*) and dedifferentiation (*Col1a1*) markers over 21 days of ATDC5 agarose culture (*n* = 3). (G) Immunohistochemistry for type II collagen, type X, and type I collagen (in red) in 2% agarose cultures supplemented with TGFβ3 and BMP7 at Day 21, with DAPI (in blue) as nuclear counterstain. Student *t* test, SEM. Key: *p < 0.05 versus Day 0; **p < 0.01 versus Day 0; ***p < 0.001 versus Day 0; ^*p* < 0.05 versus Day 1; ^^*p* < 0.01 versus Day 1; NO GF, no growth factor supplementation; D7, Day 7; D14, Day 14; D21, Day 21. S, superficial; M, medium; D, deep zone of the constructs. Scale bar 50 μm.

To assess the effect of 3D environment and mechanical stimulation on the differentiation of ATDC5 cells, cells were embedded in 2% w/v low melting agarose and grown in chondrogenic medium with or without growth factor supplementation for up to 21 days. It has been shown that 2% is the lowest agarose density that can withstand mechanical compression (Mauck et al. 2003; Thorpe et al. 2013), and compressive parameters have previously been characterized for agarose gels of different stiffness (Scandiucci de Freitas et al. 2006). Moreover, short period cultures in agarose have been previously used to maintain primary chondrocyte phenotype (Benya and Shaffer 1982; Charles Huang et al. 2004). Metal molds were designed to provide construct constriction (Thorpe et al. 2013) and mimicked the perichondrium/periosteum collar around the developing cartilage while allowing free swelling of the construct surface (Figure 3A and Figure S1B). Cells embedded in agarose had a rounded appearance reminiscent of primary chondrocytes and produced ECM rich in collagen and proteoglycans (Figure 3B,C). As mammalian cells lack agarase (Ng et al. 2009; Solursh 1991), culturing the cells in agar hydrogel resulted in largely pericellular ECM deposition which increased with time in culture. In particular, proteoglycan deposition increased significantly in constructs without growth factor supplementation and in constructs supplemented with TGFβ3 and BMP7, while sGAG levels were lower in constructs cultured in the presence of BMP7 alone at Day 21, compared with unsupplemented samples (Figure 3C). Cell metabolic activity and proliferation increased over time for all culture conditions (Figure 3D and Figure S2B). Apoptosis levels were not changed in any of the conditions (Figure 3E). Similarly to what we observed in the pellet cultures, qRT‐PCR analysis (Figure 4A–F) showed that *Sox9* was dramatically increased in the agar cultures supplemented with TGFβ3 and BMP7, compared with the unsupplemented constructs at Day 21 and to the TGFβ3 and BMP7 samples at Day 7. *Sox9* also increased in samples without growth factor supplementation but did not increase in constructs cultured in BMP7 or TGFβ3. *Col2a1* expression and *Col10a1* (marker of chondrocyte hypertrophy) was increased at Day 21 in all culture conditions. *Col1a1* expression was lower than in 2D cultures for all conditions apart from Day 21 in the unsupplemented constructs which showed no difference. Expression of *Acan* (aggrecan, the main proteoglycan in cartilage) was increased from Day 7 to Day 21 in unsupplemented samples and dramatically increased in the presence of TGFβ3 and BMP7. *Ihh* expression was increased in all samples compared with Day 0 and increased from Day 7 to Day 21 in unsupplemented and in TGFβ3 and BMP7 supplemented samples. In conclusion, similar to the pellet culture, a combination of BMP7 and TGFβ3 appeared most advantageous for the enhanced expression of chondrogenic markers and production of cartilage ECM. Immunohistochemistry confirmed deposition of type II collagen in the agar lacunae and undetectable type I collagen staining in the constructs cultured for 21 days in the presence of TGFβ3 and BMP7. Type X collagen was also detected in the constructs indicating terminal differentiation (Figure 4G).

**Figure 5:**
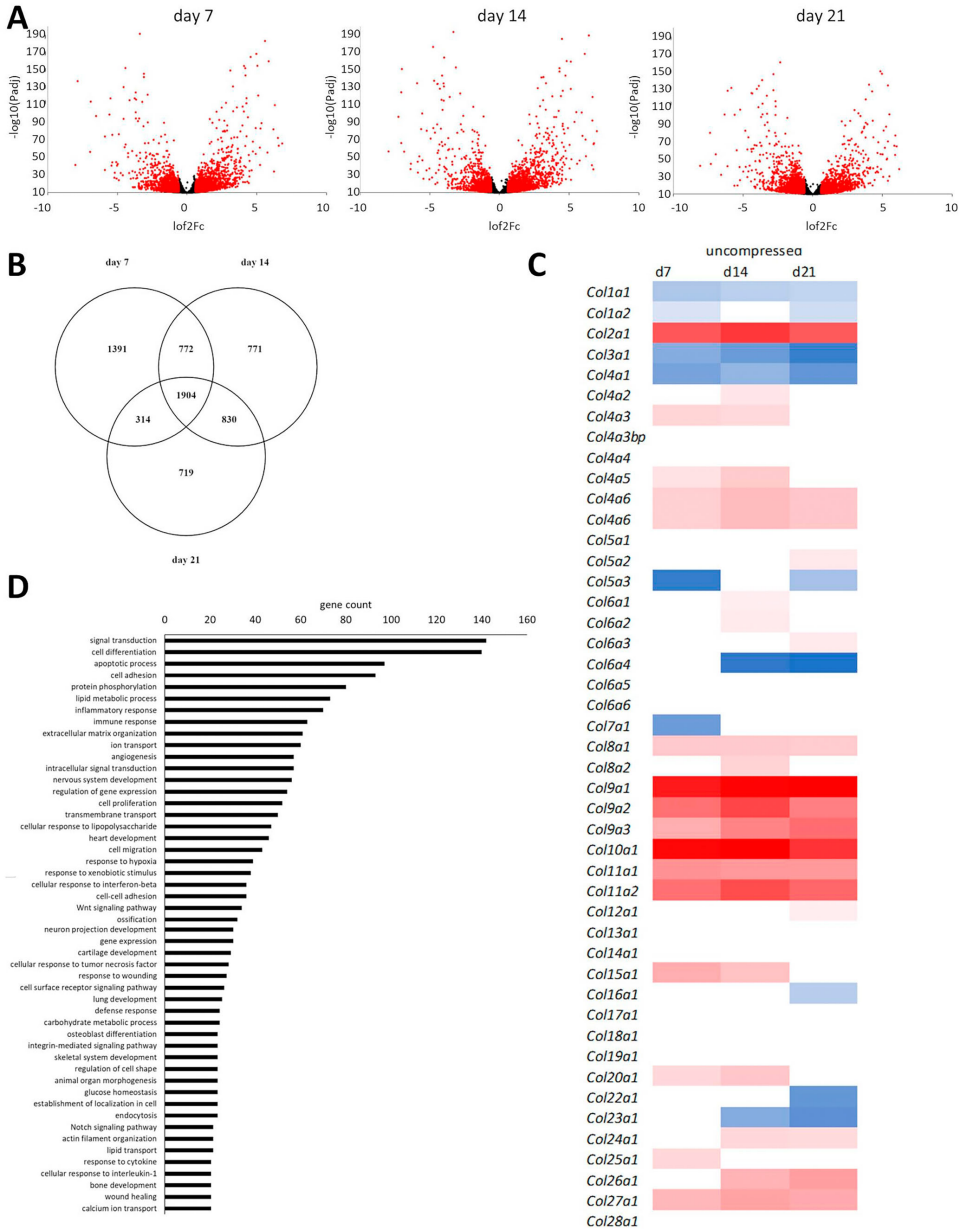
(A) Volcano plots showing differential gene expression (in red) at Day 7, Day 14, and Day 21 of culture in 2% low melting agarose with 10 ng/mL TGFβ3 and BMP7, compared with Day 1 controls. (B) Venn diagram of differentially expressed genes, showing a high overlap between the experimental timepoints. (C) Heat map of collagen gene expression in the ATDC5 cells cultured in 2% agarose constructs with 10 ng/mL TGFβ3 and BMP7 supplementation, showing an increase in cartilage specific collagens. (D) GO terms and the related gene counts pertaining to the differentially expressed genes shared between Day 7, Day 14, and Day 21 analysis. Key: d7, Day 7; d14, Day 14; d21, Day 21.

RNA sequencing was performed to elucidate the pathways triggered by encasing the ATDC5 cells in agarose while cultured with BMP7 and TGFβ3 supplementation for 21 days. There were 4053 differentially expressed genes in Day 7 samples, 3931 at Day 14, and 3544 differentially expressed genes at Day 21 compared with Day 1 controls (Figure 5A,B). A large percentage of the downregulated and upregulated genes were shared between the experimental day points, indicating that the agarose culture itself had a profound and prolonged effect on the ATDC5 biology. Several collagen genes associated with cartilage development, including *Col2a1*, *Col9a1*, *Col9a2*, *Col9a3*, *Col10a1*, *Col11a1* were increased in the constructs, while markers of fibroblasts and chondrocyte dedifferentiation (*Col1a1, Col1a2, Col5a3*) were downregulated (Figure 5C). Among the top 50 upregulated genes at all time points were the cartilage genes *Col2a1*, *Col9a1*, *Col9a2*, *Col10a1*, *Colgalt2*, and *Dcn*. Other cartilage related genes such as *Matn3*, *Comp*, and *Acan* were also highly upregulated (> 4‐fold). The GO terms for the differentially expressed genes shared between the experimental points included signal transduction, cell differentiation, cell adhesion, ECM organization, cell proliferation, response to hypoxia, WNT signaling, cartilage development, endocytosis, calcium ion transport, and integrin‐mediated signaling (Figure 5D). *Wnt5b* and *Wnt11* implicated in chondrogenesis and expressed by the prehypertrophic chondrocytes (Bradley and Drissi 2011) were also increased in the agarose cultures. Mechanoresponsive IHH signaling appeared active in the ATDC5 cells grown in the agarose constructs, with upregulation of *Smo*, *Gli1*, and *Gli2*. *Pth1r* and *Pthlh* were also upregulated, suggesting a functioning signaling loop. Moreover, *Fgf21*, shown to alleviate senescence, apoptosis, and ECM degradation in osteoarthritis via the SIRT1‐mTOR signaling pathway (Lu et al. 2021), was upregulated in the agarose encased cells, together with *Fgf1* and *2*, markers of human fetal growth plate cartilage, and *Fgfr*3, a key regulator of chondrogenesis.

### 3.3 Dynamic Compression Further Improves ATDC5 Chondrogenesis in 2% Agarose Hydrogel

**Figure 6:**
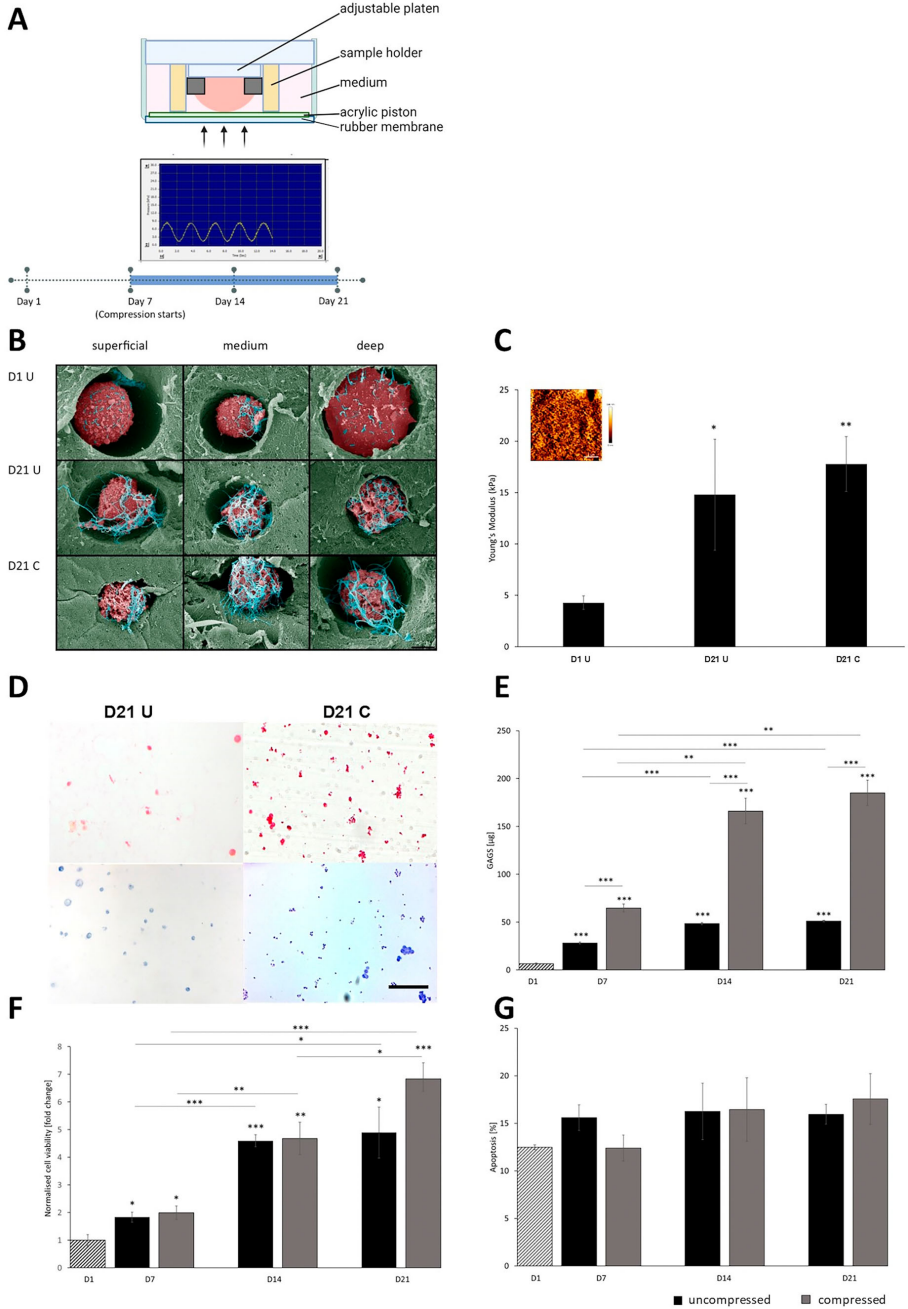
(A) Schematic drawing of the experimental set up for the compression regime, with hydrogel samples placed in the sterile BioPress® compression cell culture plates and subjected to 30 min/day sinusoid compression at 10 kPa, 0.33 Hz strain. Compression started on Day 7 of culture and continued daily until Day 21. (B) False‐colored scanning electron microscopy (SEM) images of ATDC5 cells (red) grown in 2% low melting agarose hydrogel (green), showing increased deposition of pericellular material (blue) with time of culture. Scale bar 5 µm. (C) Atomic force microscopy (AFM) measurement of the tissue nanostiffness showing an increase in tissue stiffness on Day 21 samples compared with Day 1 controls. Inset: representative AFM height image at Day 21 (*n* = 3). (D) Histological assessment of the 2% agarose constructs at Day 21 using Picrosirus Red (collagen deposition) and Toluidine Blue (proteoglycan deposition), showing increased pericellular staining in the samples subjected to daily dynamic compression. Scale bar 100 µm. (E) Quantification of the sGAG deposition using DMMB assay, showing increased proteoglycan deposition in the compressed samples when compared with the uncompressed controls at all timepoints (*n* = 3). (F) MTT assay showing increased metabolic activity over the time of culture for both uncompressed and compressed constructs (*n* = 3). (G) TUNEL assay showing a decrease in apoptosis at Day 21 in both compressed and uncompressed samples (*n* = 3). Student *t* test, SEM. Key: D7, Day 7; D14, Day 14; D21, Day 21. U, uncompressed; C, compressed. *p < 0.05; **p < 0.01; ***p < 0.001.

Under physiological conditions, articular cartilage is exposed to shear stress and compressive forces that serve as a stimulus to remodel the ECM and regulate chondrogenic differentiation (Bougault et al. 2009; Jutila et al. 2015). In hyaline cartilage, pericellular matrix (PCM) serves as a biomechanical and biochemical transducer for the chondrocytes (Wilusz et al. 2014), and previous studies of dynamically compressed chondrocytes in agarose hydrogels also show higher deposition of sGAG upon compression (Anderson and Johnstone 2017; Kelly et al. 2006; Tsuang et al. 2008). It's been previously shown that compression at 3%–10% strain and frequency of 0.17–0.5 Hz for up to 12 h leads to anabolic responses in cartilage, whereas higher frequencies and load strain and duration induce catabolic changes (Nicodemus and Bryant 2010). Human striking gait when walking is in the range of 0.2–1 Hz; therefore, for our dynamic compression experiments, we have chosen the 0.33 Hz frequency to mimic physiological conditions (Long and Srinivasan 2013; Nilsson and Thorstensson 1987). To test whether ATDC5 cells can be used to model mechanical impacts seen in native cartilage, 2% w/v low melting agarose constructs seeded with ATDC5 cells were cultured for 21 days with BMP7 and TGFβ3 supplementation and subjected to dynamic compression for 30 min daily starting on Day 7 of culture. A cross‐section of a well of the BioPress® compression plate containing a sample and placed in the Flexcell FX5000™ Compression System is shown on the schematic in Figure 6A. Interestingly, SEM imaging showed increased accumulation of pericellular material in the uncompressed and compressed constructs at Day 21 compared with the Day 1 controls (Figure 6B). This had an impact on the overall tissue stiffness which showed a nanoscale Young's modulus of > 15 kPa at Day 21, comparable with native mouse cartilage (Stolz et al. 2009) (Figure 6C). Dynamic compression led to a gradual increase in pericellular deposition of collagen and proteoglycan in the compressed samples compared with the uncompressed controls (Figure 6D,E). Our low impact compression regime had no effect on the metabolic activity of cells (Figure 6F), which increased throughout culture in both compressed and uncompressed constructs. However, proliferation increased in Day 7 compressed samples compared with the uncompressed controls (Figure S2C). There was no difference in apoptosis between the compressed and uncompressed samples (Figure 6G).

**Figure 7:**
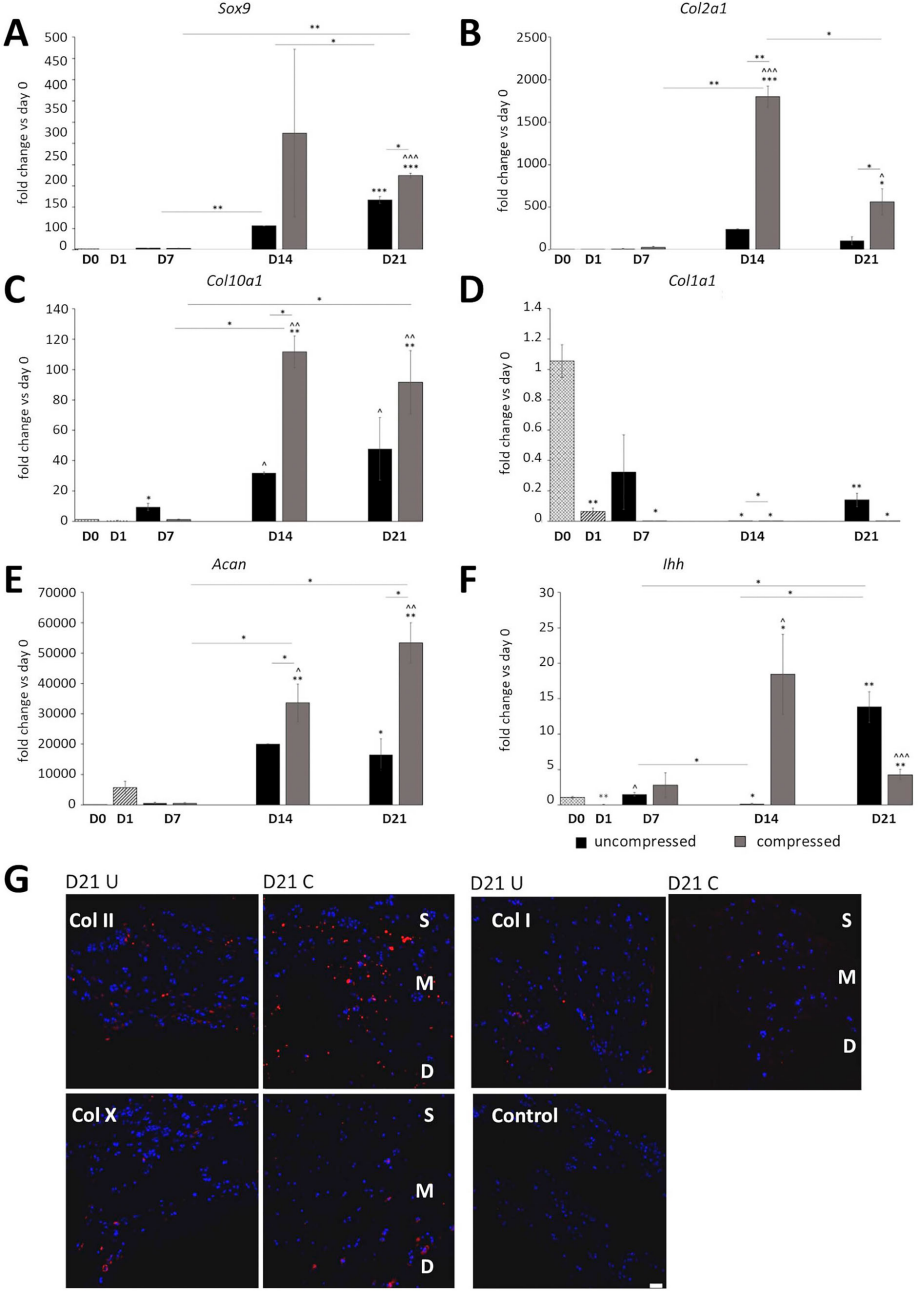
(A–F) qRT‐PCR of chondrogenesis (*Sox9*, *Col2a1*, *Acan*), differentiation (*Col10a1*, *Ihh*) and dedifferentiation (*Col1a1*) markers over 21 days of ATDC5 agarose culture with and without compression (*n* = 3). (G) Immunohistochemistry for type II collagen, type X, and type I collagen (in red) in 2% compressed and uncompressed agarose cultures at Day 21, with DAPI (in blue) as nuclear counterstain. Student *t* test, SEM. Key: *p < 0.05 versus Day 0, **p < 0.01 versus Day 0, ***p < 0.001 versus Day 0, ^*p* < 0.05 versus Day 1, ^^*p* < 0.01 versus Day 1, ^^^*p* < 0.001 versus Day 1, D7, Day 7; D14, Day 14; D21, Day 21. S, superficial; M, medium; D, deep zone of the construct. U, uncompressed; C, compressed. Scale bar 50 μm.

Dynamic compression increased the expression of chondrogenic markers (Figure 7A–F), including *Sox9* at Day 21, *Col2a1* and *Acan* at Day 14 and Day 21, and *Col10a1* at Day 14 when compared with the uncompressed controls. Type I collagen (*Col1a1*) expression was much lower in the compressed samples than the uncompressed controls, and significantly lower than Day 0 in all samples. *Ihh* expression increased in the uncompressed samples at Day 21, and in the compressed samples followed a pattern similar to the *Sox9* expression. Immunohistochemistry for type I, II, and X collagen confirmed the RT‐qPCR results and showed an increase in type II collagen deposition upon compression. Moreover, type X collagen was increased in the compressed samples suggesting terminal differentiation (chondrocyte hypertrophy), while type I collagen staining was decreased in the compressed samples (Figure 7G).

Daily dynamic compression led to increasingly differential gene expression in the 2% agarose constructs. There were 3866 differentially expressed genes in Day 7 compressed samples (one instance of compression) compared with Day 1 controls; 3927 in Day 14 compressed samples, and 4246 in Day 21 compressed samples. A large percentage of these was shared between the experimental timepoints, indicating a persisting effect of agarose encapsulation on ATDC5 phenotype (Figure S3A,B). Similar to the uncompressed samples, among the 50 highest expressed genes in the compressed samples at Day 7, 14, and 21 compared with Day 1 controls were the cartilage genes: *Col2a1*, *Col9a1*, Col9a2, *Col10a1*, *Colgalt2*, and *Dcn. Acan, Comp, Matn1*, and *Matn3* were also upregulated > 4‐fold (Figure S3C). GO terms in the compressed cells at Day 21 compared with Day 1 included multicellular organism development, cell proliferation, ECM organization, cartilage development, cell‐matrix adhesion, and collagen fibril organization. Thirty‐eight percent of GO terms were shared between the differentially expressed genes in the uncompressed and compressed samples at Day 21 compared with Day 1 controls, and included cartilage development, ECM organization, integrin‐mediated signaling, cell‐matrix adhesion, and canonical Wnt signaling, indicating the profound effect that the 3D environment had on chondrogenesis. GO terms exclusive to the compressed samples included: cell response to hypoxia, Notch signaling, chondrocyte differentiation, bone development, circadian rhythm, intracellular signal transduction, lipid metabolism, actin filament organization, collagen biosynthesis, and response to mechanical stimulus.

**Figure 8:**
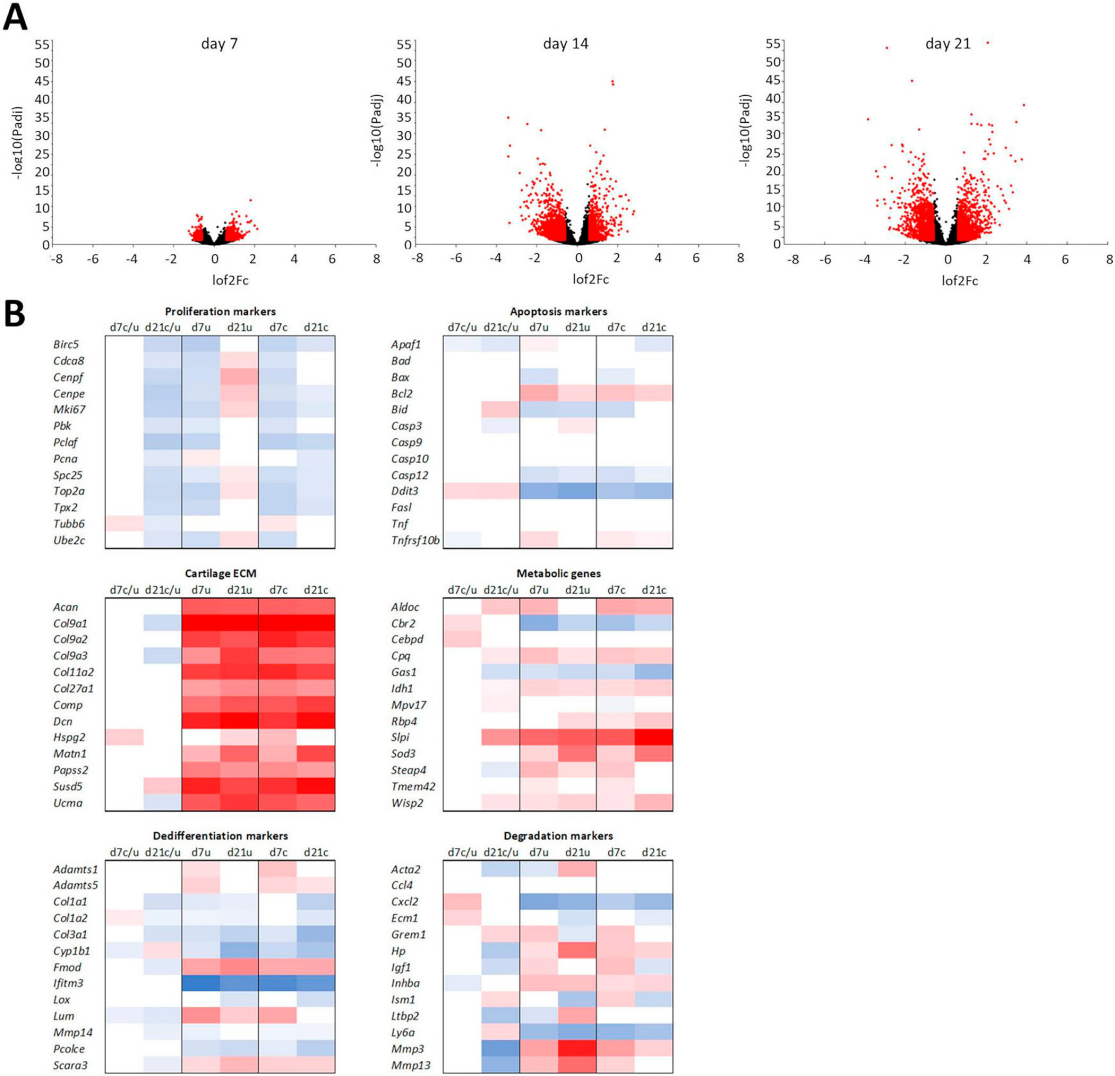
(A) Volcano plots showing differential gene expression (in red) at Day 7, Day 14, and Day 21 of culture in 2% low melting agarose with daily dynamic compression, compared with uncompressed controls. (B) Heat map of gene expression of markers of early proliferative chondrocytes, apoptotic markers, markers of cartilage ECM, metabolic genes, and markers of dedifferentiation and cartilage degradation (Chen et al. 2022). Key: d7, Day 7; d14, Day 14; d21, Day 21; c, compressed; u, uncompressed.

To visualize the direct effect of dynamic compression on the temporally resolved differentiation of ATDC5 cells embedded in 2% agarose, a comparison was performed between the compressed and uncompressed samples at individual day points (Figure S4A). RNA sequencing revealed gradual increase in the expression of mechanoresponsive genes throughout the experiment (Figure 8A). Daily dynamic compression stimulated genes and pathways that largely related to intracellular calcium, mechanosensing, chondrogenesis, ECM deposition, and WNT signaling. At Day 7 (one instance of compression), there were 881 differentially expressed genes in the compressed samples compared with uncompressed, representing an immediate response to a mechanical stimulus. The GO terms for these included regulation of transcription, translation, regulation of cell cycle, and mitochondrial respiration. Among the upregulated mechanoresponsive genes were tubulin genes *Tub1a1* (upregulated 1.6 fold) and *Tubb2a* (1.7 fold) indicating cytoskeletal reorganization, the chondrocyte‐specific transcription factor *Sox9* (upregulated 1.65 fold), *Lrp1* (upregulated 1.6 fold) important for maintaining cartilage homeostasis, hedgehog signaling transcription factor *Gli1* (upregulated 2.1 fold), and the ion channel *Trpv4* (upregulated 1.4 fold). 2044 genes were differentially expressed between the compressed and uncompressed samples at Day 14, and pertained to differentiation, cell migration, proliferation, apoptosis, cartilage development, ECM organization, and regulation of TGFβ and FGF signaling pathways. 2538 differentially expressed genes at Day 21 related to transcription, translation, lipid metabolism, regulation of cell cycle, and WNT signaling (Figure 8B). *Bmp6* implicated in regulation of proteoglycan synthesis by the chondrocytes (Bobacz et al. 2003) was increased 1.6‐fold, *Fzd5* able to maintain proliferation of mesenchymal stem cells and prevent senescence was increased 1.8‐fold, *Grem1*, a cartilage progenitor marker was increased 1.8‐fold and noggin (*Nog*) shown to prevent osteoarthritis progression in rats was increased 1.9‐fold. Moreover, compressed samples presented an increase in the markers of cartilage repair, including *Hmox1* and *Wnt5b*. Several markers of cartilage degradation (*Col1a1* (2.1‐fold), *Il1r1* (5.1‐fold), *Mmp3* (10.9‐fold), *Mmp9* (2.3‐fold), *Mmp13* (6.4‐fold), *TnC* (7.5‐fold), and *Wnt5a* (3‐fold)) were also decreased in the compressed samples compared with the uncompressed controls.

Overall, agarose encapsulation appeared to reduce the expression of markers of early, proliferative immature chondrocyte (Chen et al. 2022), which then increased again in samples without compression over the 21 days of culture and decreased under prolonged dynamic compression. Agarose encapsulation led to an upregulation of antiapoptotic proteins and increased ECM production. Interestingly, while the differences in cartilage ECM genes were not pronounced between the compressed and uncompressed samples, the expression of dedifferentiation and cartilage degradation markers was decreased in the samples cultured under dynamic compression (Figure 8B) (Chen et al. 2022). These changes suggest that encapsulation of the ATDC5 cells in agarose dramatically improves chondrogenesis and cartilage ECM production and decreases apoptosis, while daily dynamic compression stimulates metabolic activity and downregulates dedifferentiation and ECM degradation (Figure S4B).

## 4 Discussion

Cartilage is a zonally stratified mechanoresponsive tissue which is difficult to replicate in a laboratory setting. Recent advances in tissue engineering are opening new possibilities to develop models of cartilage that facilitate in vitro studies of cartilage biology. This project aimed to improve the commonly used model of chondrogenesis, utilizing the ATDC5 cell line, by providing a three‐dimensional environment to support cartilage development in vitro, and to create an easy to manufacture mechanoresponsive model that could be applied in the study of skeletal diseases. In our study, we decided to employ BMP7 and TGFβ3 growth factors to augment ATDC5 chondrogenesis in both 3D culture systems. TGFβ‐family proteins, and specifically TGFβ3 and TGFβ1, are routinely used to support skeletal cells and differentiation of bone‐marrow derived stem cells in in vitro studies. They have both been shown to support chondrogenesis of various skeletal progenitor cells with varied effects on cell proliferation (James et al. 2009; Wee et al. 2022). TGFβ3 is more commonly used of the two in the context of chondrogenesis and cartilage tissue engineering and has been previously used to look response of mesenchymal stem cells to dynamic compression (Thorpe et al. 2010). BMP7 has been shown to promote chondrogenesis of human amniotic epithelial cells (Zhou et al. 2011) and was chosen due to its ability to decrease the fibrocartilage phenotype in cultured human articular chondrocytes (HACs) (Charles Huang et al. 2004; Huang et al. 2018; Ripmeester et al. 2021; Thorpe et al. 2010). Indeed, similarly to what was previously found for the osteoarthritis‐derived human chondrocytes (HACs), the supplementation with TGFβ3 and BMP7 combined dramatically improved ATDC5 chondrogenesis (Figures 1, 2, 3, 4). We have shown that ATDC5 pellets cultured with a combination of BMP7 and TGFβ3 increased in size, with cells presenting with rounded chondrocyte‐like morphology, increased metabolic activity and ECM deposition (Figures 1 and 2) (Caron et al. 2012; Lahm et al. 2010). We have also shown for the first time that 2% w/v low melting agarose is supportive of ATDC5 chondrogenesis with cells presenting with rounded morphology characteristic of chondrocytes, and with larger cells occupying the deeper zones of the constructs and type X collagen (marker of chondrocyte hypertrophy, (Han et al. 2024; O'Keefe et al. 1994)) expression suggesting terminal differentiation (Figures 3, 4, 6 and 7). Three‐Dimensional culture systems such as pellets, sponges, and hydrogels have been shown to support the differentiation state and survival of several skeletal lineage cells (Kelly et al. 2006; Tare et al. 2005; Thorpe et al. 2013; Watts et al. 2013; Wilhelm et al. 2020). Interestingly, RNA sequencing of our 2% agarose constructs seeded with ATDC5 cells revealed a large number of genes that were differentially expressed upon encapsulation and remained upregulated throughout culture, comprising genes involved in cartilage development, ECM organization, ossification, and WNT signaling. Expression of several components of WNT, FGF, and HH pathways were upregulated, indicating that agarose encapsulation improved chondrogenic differentiation of ATDC5 cells. Moreover, the deposition of pericellular ECM increased over time in our compressed constructs compared with the uncompressed controls (Figures 5 and 6). Dynamic compression of the agar embedded ATDC5 cells promoted better chondrogenesis and decreased dedifferentiation, and appeared to reduce expression of genes associated with osteoarthritis, injury, and catabolism while increasing expression of *Fgf21* known to alleviate senescence and ECM degradation in OA (Lu et al. 2021). WNT signaling was also modulated by compression, with *Wnt5a*, responsible for skeletal outgrowth during development but also inducing OA catabolism gradually decreased in the compressed samples (De Santis et al. 2018; Huang et al. 2017) while *Wnt5b*, promoting chondrogenesis and cell migration (Bradley and Drissi 2011), gradually increased. Moreover, several mechanoresponsive genes including *Trpv4* and other genes involved in ion transport, planar polarity, signal transduction, and response to mechanical stimulus were differentially expressed, and the overall transcriptomic profile of the compressed samples showed a decrease in dedifferentiation and degradation markers and increase in metabolism and cartilage ECM production (Figures 7 and 8). In comparison to the 2D culture, 3D pellets, and unsupplemented agarose constructs, the ATDC5 cells cultured with BMP7 and TGFβ3 supplementation and with dynamic compression showed improved proteoglycan deposition, metabolic activity, increased expression of chondrogenesis and cartilage differentiation markers and dramatically reduced expression of type I collagen (Figure S4B).

These promising results highlight the potential of hydrogel‐enhanced chondrogenesis and propose an easy to manufacture, adaptable and scalable in vitro model to study mechanoresponses, intracellular signals, and PCM involvement in cartilage development and disease. We have shown that ATDC5 cells cultured in our system express markers of all stages of chondrogenesis. Moreover, agarose gels show a high degree of transparency which lends itself to live imaging and confocal microscopy approaches. However, the main limitation of this system is that mammalian cells do not express agarase (Ng et al. 2009), and therefore, the ECM deposition and remodeling is largely happening in the pericellular niche. In the future, several of the parameters established in this study can be extrapolated to other hydrogel‐based systems (e.g., collagen and/or hyaluronic‐acid based hydrogel models, where ECM remodeling is possible (Kowalski et al. 2022; Long et al. 2022; Sun et al. 2023)) to further optimize cartilage tissue engineering approaches. The zonal stratification could also be further enhanced using gradients in gel stiffness (Zhu et al. 2018), stratified growth factor stimulation (e.g., BMP7 supplementation increased *Col10a1* levels in ATDC5 pellets, and differential TGFβ3 and BMP4 supplementation has been shown to drive differentiation of human embryonic and iPS cells into more articular or mature/hypertrophic phenotype, respectively (Richard et al. 2023)) and/or bioprinting specific microniches with respective growth factor cocktails (Dimaraki et al. 2021).

To conclude, ATDC5 cells grown in 2% agarose with BMP7 and TGFβ3 growth factor supplementation and cyclic dynamic compression undergo chondrocyte‐like differentiation, induce expression of mechanoresponsive genes and present a good model to study the role of pericellular ECM components and mechanosensing in healthy and diseased cartilage.

## Author Contributions

**Marc V. Farcasanu:** investigation, writing – original draft, writing – review and editing. **Thais de las Heras Ruiz:** investigation. **Francesca M. Johnson de Sousa Brito:** investigation, data curation and analysis. **Jamie Soul:** data curation and analysis. **Jonathan Coxhead:** data curation and analysis. **Matthew J. German:** supervision. **David A. Young:** conceptualization, supervision, writing – review and editing. **Ana M. Ferreira‐Duarte:** conceptualization, supervision, writing – review and editing. **Katarzyna A. Piróg:** conceptualization, supervision, writing – original draft, writing – review and editing.

## Conflicts of Interest

The authors declare no conflicts of interest.

## Supporting information


**Supporting information**.

**Supporting information**.

**Supporting information**.

**Supporting information**.

**Supporting information**.

**Supporting information**.

## Data Availability

The data that support the findings of this study are openly available in Gene Expression Omnibus at https://www.ncbi.nlm.nih.gov/geo/, reference number GSE280260.
