# Supplementary figures and images for: Dynamic Compression Improves Chondrogenesis in the Tissue Engineered Model of Cartilage
Source: Biotechnol Bioeng. 2025 May 25;122(9):2574–91. doi: 10.1002/bit.29026 (PMC12322637; doi:10.1002/bit.29026)

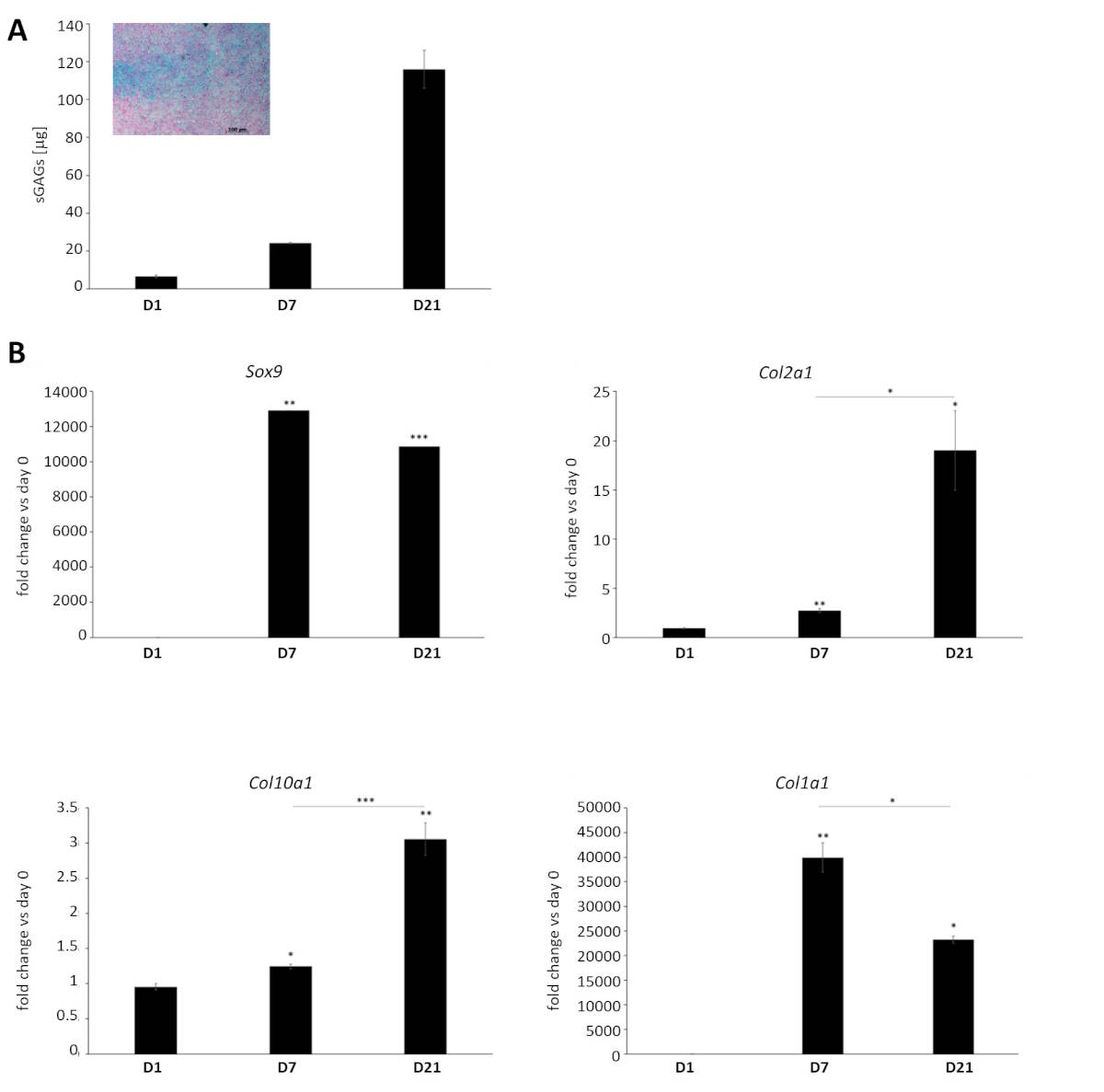


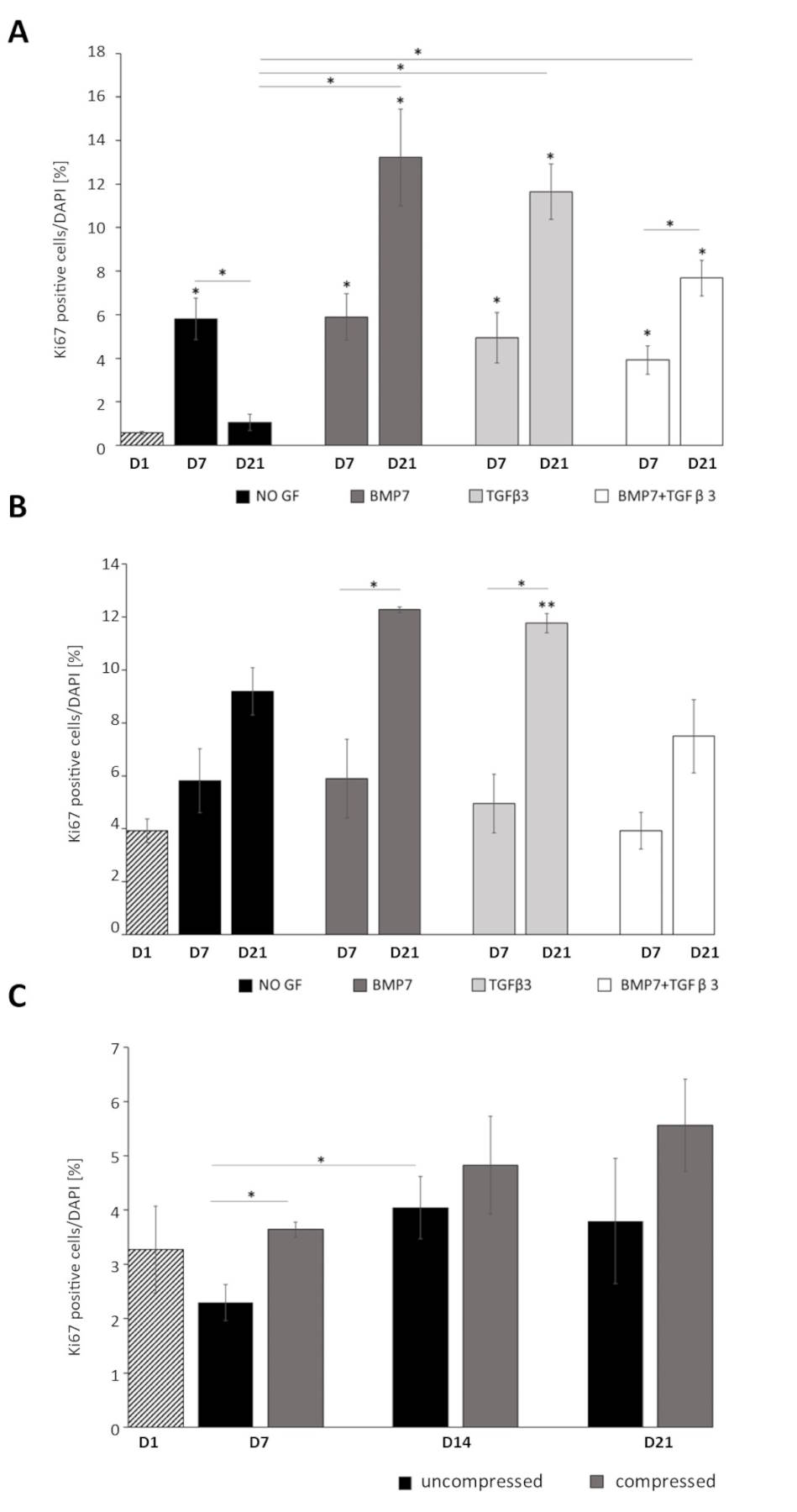


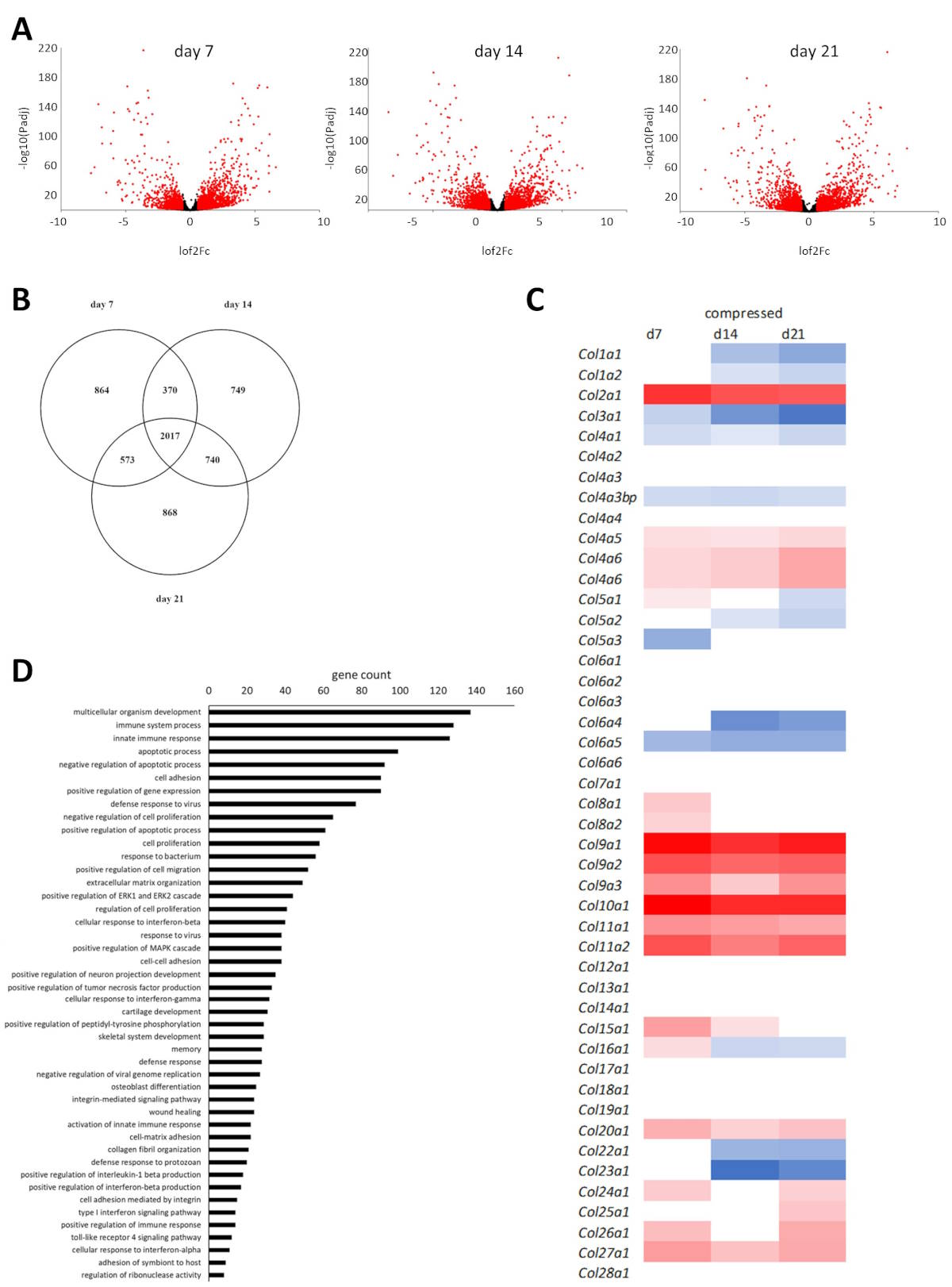

Supplement: Supplementary file 1 — Supporting information. [file BIT-122-2574-s004.docx]

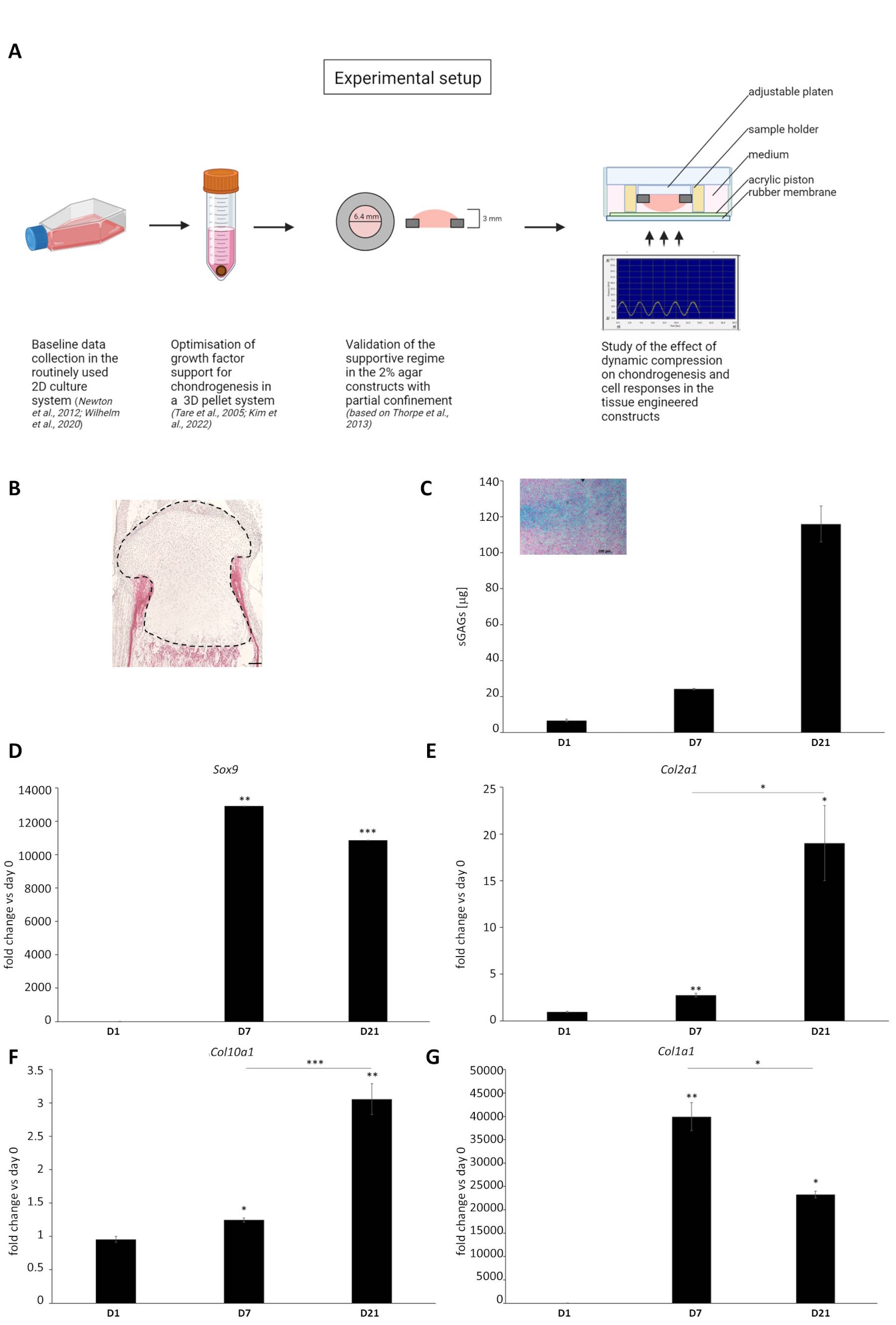

Supplement: Supplementary file 2 — Supporting information. [file BIT-122-2574-s002.jpg]

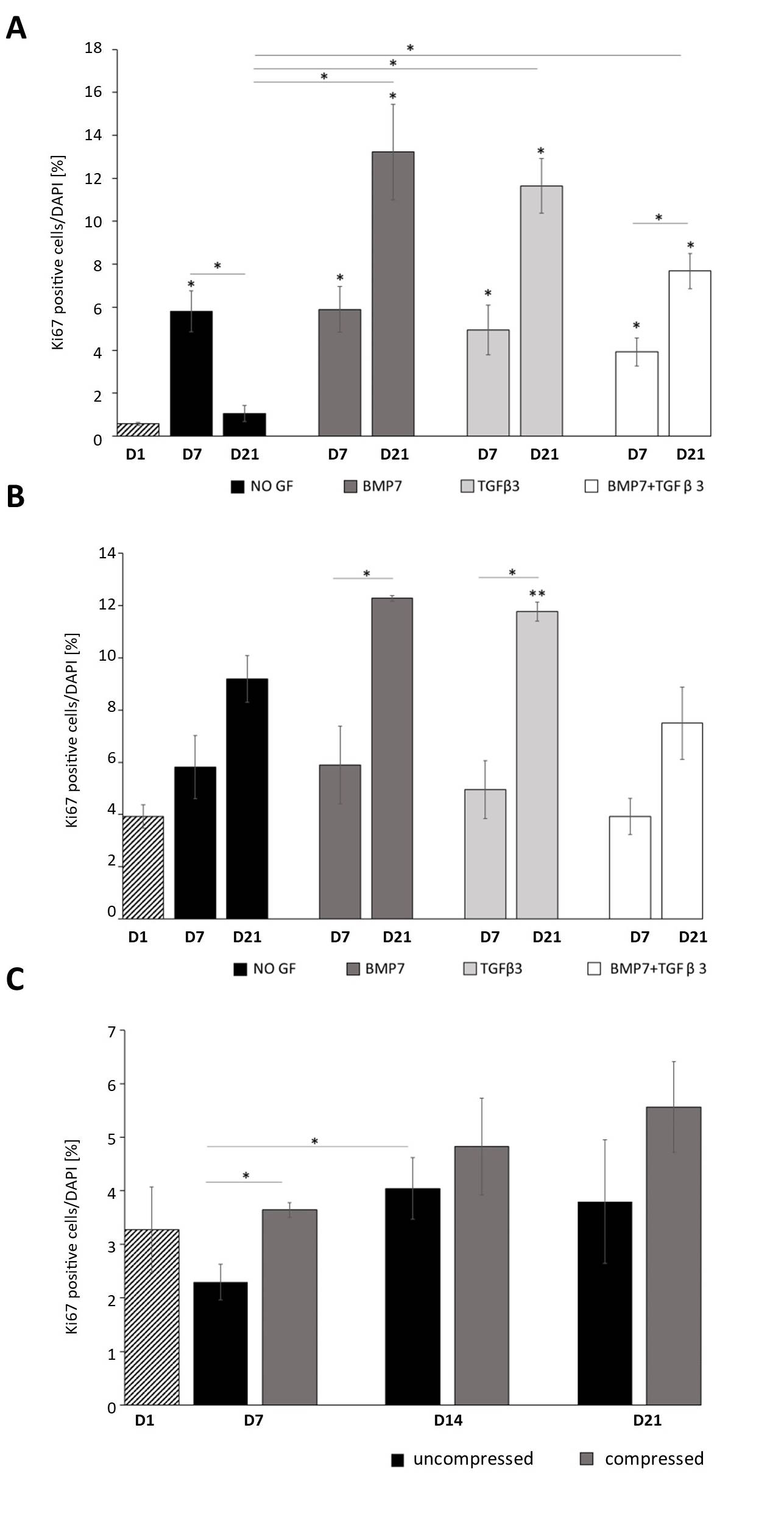

Supplement: Supplementary file 3 — Supporting information. [file BIT-122-2574-s003.jpg]

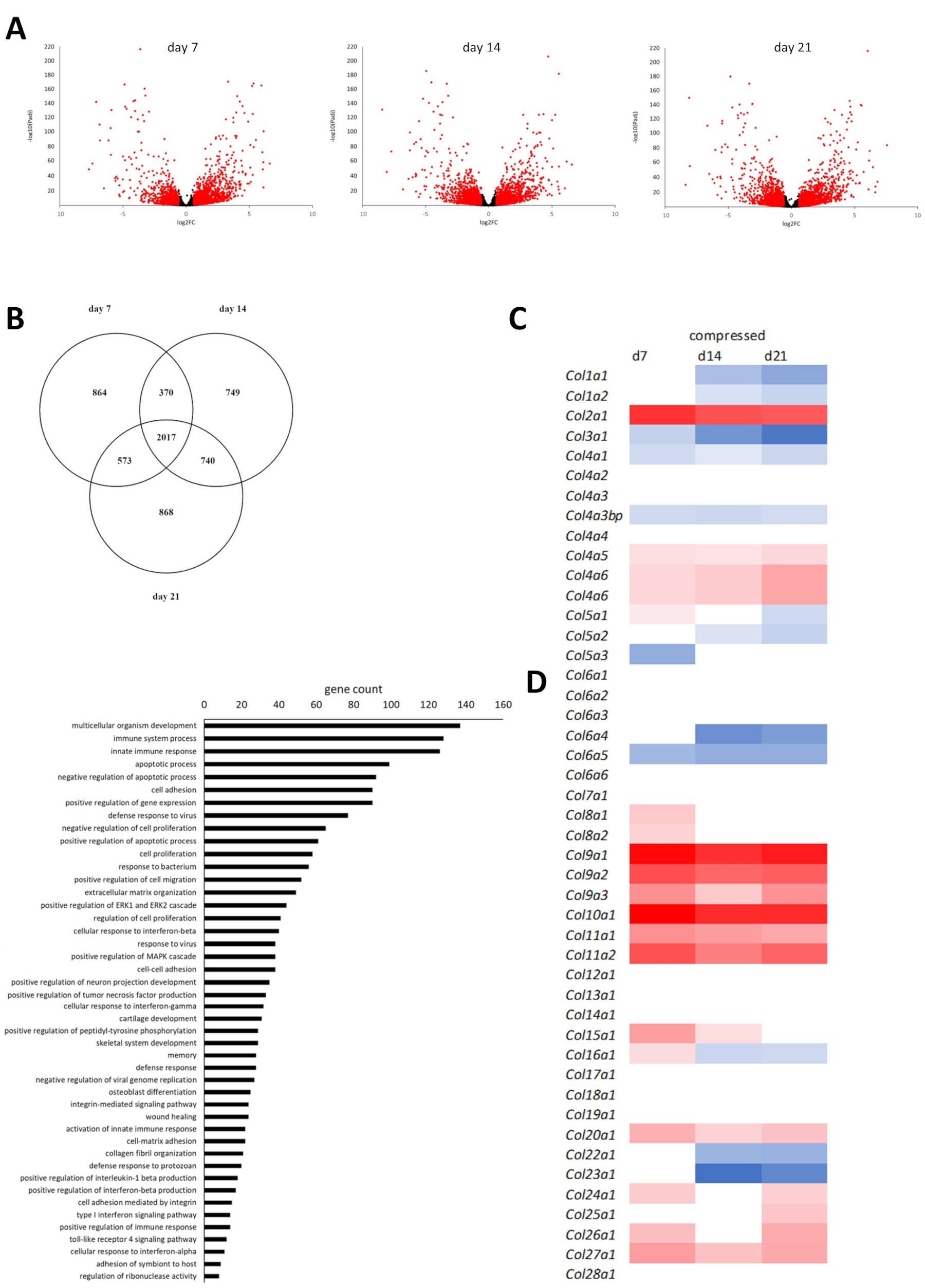

Supplement: Supplementary file 4 — Supporting information. [file BIT-122-2574-s001.jpg]

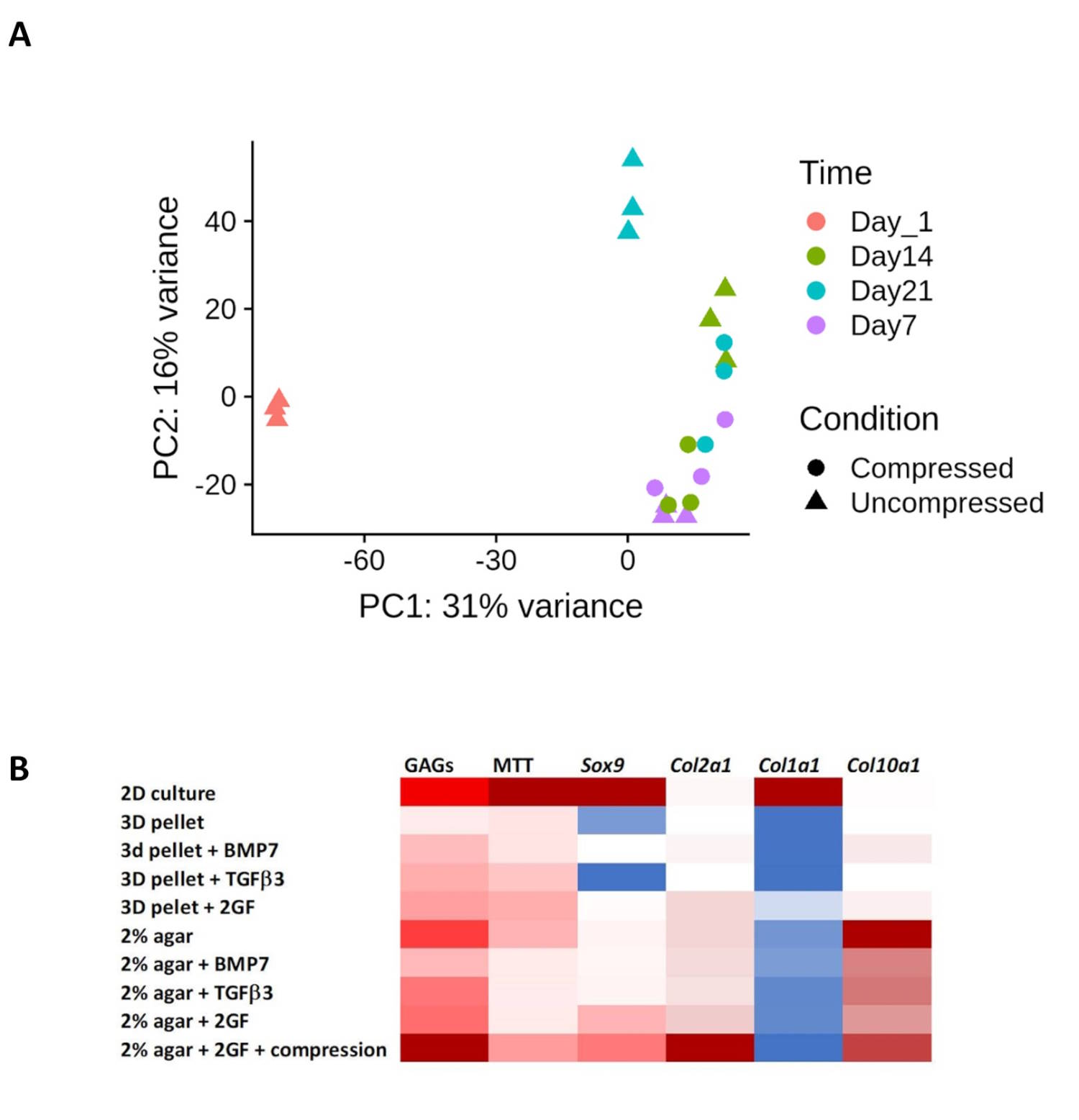

Supplement: Supplementary file 5 — Supporting information. [file BIT-122-2574-s006.jpg]
