# Supplementary material for: Dynamic Compression Improves Chondrogenesis in the Tissue Engineered Model of Cartilage
Source: Biotechnol Bioeng. 2025 May 25;122(9):2574–91. doi: 10.1002/bit.29026 (PMC12322637; doi:10.1002/bit.29026)
Supplement: Supplementary file 6 — Supporting information. [file BIT-122-2574-s005.docx]

Supplemental Table 1 – Primer sequences (5’-3’).

| **Gene** | **Forward primer** | **Reverse primer** |
| --- | --- | --- |
| *Col1a1* | CATGTTCAGCTTTGTGGACCT | GCAGCTGACTTCAGGGATGT |
| *Col2a1* | TGGTAACCCAGGGACTGATG | CCAGCAATTCCAGGAGCA |
| *Col10a1* | GCATCTCCCAGCACCAGA | CCATGAACCAGGGTCAAGAA |
| *Acan* | TGAAGCAGAAGGTCTGGACA | CCAGAAGGAATCCCACTAACA |
| *Ihh* | TGCATTGCTCTGTCAAGTCTG | GCTCCCCGTTCTCTAGGC |
| *Sox9* | GTACCCGCATCTGCACAAC | CTCCTCCACGAAGGGTCTCT |
| *18S* | GCCGCTAGAGGTGAAATTCTT | CGTCTTCGAACCTCCGACT |
